# Challenges in the Management of Home-Based Care Experienced by a Caregiver with Autism Spectrum Disorder

**DOI:** 10.1089/pmr.2021.0043

**Published:** 2021-11-17

**Authors:** Sayaka Ohsawa, Hisashi Yoshimoto, Ryo Ohsawa, Satoko Takahashi, Shoji Yokoya

**Affiliations:** ^1^ Kitaibaraki Center for Family Medicine, Kitaibaraki, Japan.; ^2^ Department of Family Medicine, General Practice and Community Health, Faculty of Medicine, University of Tsukuba, Tsukuba, Japan.; ^3^ Takahashi Clinic, Saitama, Japan.

**Keywords:** autism spectrum disorder, communication supports, family caregiver, home care services, interprofessional team, palliative care

## Abstract

We describe the case of a 37-year-old woman with autism spectrum disorder (ASD) who lived with a mother with end-stage breast cancer and a visually impaired father. She was the main caregiver for her mother, who was receiving home-based palliative care. The caregiver needed training on communication and task management so that she could manage the communication with home care staff and perform more house chores. It was also necessary to share information with home care staff about ASD and how to treat her with understanding and respect. Although most support for people with ASD focuses on schools and workplaces, to offer successful end-of-life care at home, medical and home staff need to understand and communicate well with people with ASD and provide multiple types of support. Research, guidebooks, and visuals about main caregivers who have ASD and improving the inclusivity among staff members are necessary for providing successful home care and meeting caregivers' and patients' needs and expectations.

## Introduction

Autism spectrum disorder (ASD) is now a widely recognized disorder and the overall prevalence among adults across countries and ethnic groups is reported to be 1.1%.^1^ Although social support systems for school, employment, and parents have improved, later life events may bring about the need for other types of social support and understanding among those around people with ASD. Without this change, physical and mental health and social adaptation would be adversely affected. Aging of supporters for people with ASD, for example, their parents and siblings, is inevitable. An example of a later life event is declining health and death of the parents, which would negatively influence the overall health of a person with ASD. Not all medical and home care staff members fully understand the traits of ASD; therefore, miscommunication could easily happen between the staff members and people with ASD who are caregivers for their family members, which could influence the quality of life for caregivers with ASD and their family members. We describe a case of a caregiver who successfully completed home-based end-of-life care for her critically ill mother with training in multitask management and communication skills and appropriate support by home care staff members who initially were not sufficiently familiar with ASD. The staff members needed to learn about ASD and how to communicate well with people with ASD. The knowledge and experiences gained by staff members through caring for her and her mother helped foster and boost their acceptance of and inclusivity for the person with ASD.

## Case Description

As home care doctors and general practitioners, we encountered a 37-year-old woman with ASD who lived with her parents in Japan. Her mother had end-stage breast cancer. We visited her house regularly to care for her mother. The woman with ASD was the main caregiver and a key person for her mother because her father was visually impaired. There were some obstacles we need to overcome together, but in the end, she was able to fulfill the role of the main caregiver for her mother.

When we started home care for the mother, we did not have any knowledge that the daughter was a person with ASD. However, we occasionally heard about her from home visit nurses and home care staff because they worried about whether the mother could continue to stay at home due to the lack of care provided by family members. According to home care staff members, the woman with ASD was usually not present when the home care staff visited the home for her mother's care. They could not contact her or talk with her about her mother's condition. She focused on dishwashing and did not appear concerned about how her mother's medications or clothes were organized or whether the room was clean or not. She could not communicate well with home care staff even when she talked with them. Thus, we did not initially recognize that she was a person with ASD or another developmental disorder. We were wondering why she was having trouble managing the home care schedule and chores even though she worked as an engineer. The home care staff's distrust of the daughter caused irritation and stress in the daughter and the home care staff.

After a few home care visits, with the daughter's consent, the mother's care manager informed us that she had been diagnosed with ASD in adulthood. Her parents had not been informed of the diagnosis. The daughter did not want to let her parents know about the diagnosis. The home care staff started to feel strongly that it was too difficult for the daughter to continue home care for the mother. The woman with ASD also felt difficulties in communicating with staff and understanding how her mother felt or how it feels to be severely ill. Therefore, we initiated consultation and social skill training in our outpatient clinic for the daughter. We discussed the traits of ASD as well as solutions or strategies to manage ASD traits in the home care setting. When we gave her instructions, we tried to make them more specific and provided examples so that she could understand them easily. For example, we suggested that she needed to come into her mother's room to share her mother's condition when the home care staff made a visit.

After several consultations, she gradually obtained life skills in communication, scheduling, and managing things related to home care, such as expectations during home care staff visits or what she should do for her mother. Her psychological stress and caregiving burden decreased day by day. We also shared the fact that she was diagnosed with ASD with home care staff as well as appropriate attitudes, support, and communication styles. We shared her behavioral characteristics with staff, such as lower interest in sharing emotions with others or having difficulties in understanding social cues such as eye contact, facial expressions, and metaphors.

Five months after the start of home-based care, her mother's condition worsened temporarily. The woman with ASD could not understand her mother's discomfort or pain and her father's sadness and serious feelings about the poor prognosis of his wife. She could not understand why her father was at a loss for words upon hearing the doctor's explanation of her mother's severe condition. Therefore, she asked him “Didn't you hear, Dad?” in a loud voice. These types of reactions by the daughter interfered with her father's acceptance of her mother's condition and he was unable to continue to listen the explanation. After this event, we explained why her father become silent while during the explanation of her mother's condition. We made a rule that we will tell her beforehand what we would say and how she should act when we share bad news. For example, we told her beforehand that we were going to tell her father about her mother's poor condition and prognosis and asked her to listen until we finished. We also told her how her father would react and our guess of the reasons for his reaction. Inappropriate behavior that is not suitable for the situation decreased afterward. She also felt relieved that she knew what she should do and could support her father as much as possible.

Seven months after the start of home-based care, the mother passed away at home as her mother and she herself hoped. When her mother needed to use oral opioids and subcutaneous injection of opioids, the woman with ASD was able to communicate with homecare staff and ask for help to offer better care for her mother. Her mother died peacefully without any severe pain or discomfort. The woman with ASD did not become confused and was able to accept her mother's death peacefully with her father.

## Discussion

In this case, a person with ASD was able to complete home care for her mother with end-stage cancer after receiving training in multitask management and communication skills. Sharing information about ASD and how to communicate a person with ASD with understanding and respect among home care staff fostered staff acceptance and inclusivity. For offering successful home-based care, the necessity to be more inclusive to others and reflective about ourselves as medical and home care staff member is recognized through this case.

There were two main obstacles we had to solve for maintaining home-based care for the mother. These detailed solutions for the main caregiver and home care staff are shown in Table 1.

**Table 1.** Challenges and Solutions for the Main Caregiver and Home Care Staff

Challenge	Solution for the main caregiver	Solution for home care staff
More home care tasks, such as cleaning, using medicine properly, and physical caregiving	Make to-do lists and consult with her general practitioner about priorities during an outpatient visit Take a leave of absence for caregiving	Know her traits and perceived shortcomings Ask her manager at her workplace to learn more about her traits
Gaps in expectations	Know what is generally expected of a main caregiver at home (e.g., be present during a home care nurse visit)	Learn general ASD traits and specific traits of a person with ASD When there is a gap in expectations, tell her about it precisely (e.g., nurses want to share the information about her mother and her mother's condition)
Not good at expressing her own feelings and thoughts	Do not increase stress and discuss sources of stress with her general practitioner in the consultation room	Share information with home care staff about what she wants or does not want and her requests in the consultation room and ensure that she agrees to the information to be shared
Inability to read between the lines, such as during serious conversations	Understand beforehand what she is expected to do	Tell her beforehand what would be said and how she should act when bad news is shared

ASD, autism spectrum disorder.

First, due to ASD traits, the daughter had difficulties in understanding the feeling of others, reading between the lines, multitasking, and adapting to new situations, which are usually required for good home care. We needed to give her advice about the skills and solutions for these problems at several outpatient consultations as well as during home care visits. We also needed to tell her in advance what she should do and how she should act in order not to hinder her father's understanding and acceptance when we explained the severity of her mother's condition to her father.

The second obstacle is the lack of understanding about ASD among home care staff. The home care staff developed a negative impression of the woman with ASD due to her communication style and behavioral traits, which could cause staff burnout and a premature end to home-based care. We needed to share information with other home care staff members about her communication traits, perceived shortcomings, and needs for carrying out caregiving. For home care staff, we provided information and some tips about how we treat her and communicate with her smoothly. By knowing her traits, what she is not good at, what she cares about, and what she put importance on, they could understand the reasons of her behavior and came to perceive her as not a person who were not able to complete her mother care at home but a person who were facing with one of a life tasks to care her aged and sick parents.

Life events, for example, facing disease or death of parents, are inevitable and may have a negative impact on people with ASD in terms of physical and mental health and social adaptation.^2^ However, these situations have not been extensively studied. A mismatch of what has been researched about adults with ASD in general and what is hoped to be researched about adults with ASD and their supporters has been reported, including practical, social, and mental health issues.^2,3^ In addition, a lack of experience and knowledge among medical and home care staff, which results in anxiety and stress among people with ASD and staff, has also been reported.^4^ In these situations, it would be difficult to manage the home-based care for patients and their caregivers, as in this case. Research about people with ASD who become caregivers for their family members is necessary to increase the quality of home-based care, for example, what kinds of needs or obstacles they have when they fulfill the role of a main caregiver or what medical staff members should know when providing bad news or teaching home care skills. It is also important to create guidebooks and visuals for medical or home care staff members to understand what people with ASD have trouble fulfilling the role of a main caregiver and what the situations look like for them, and the important points when offering palliative care for parents or siblings of people with ASD. Furthermore, as autistic people themselves point out the need for general practitioners' practices to become more inclusive to autistic people.^5^ To improve our inclusivity not only for people with ASD but also for those around people with ASD, all home care staff always need to reflect on ourselves whether we are understanding their individual traits well and support them properly and accordingly, or we are making up emotional barriers without realizing them.

Most adults with ASD or developmental disorders live with their parents or siblings in adulthood. They may be the main caregivers for their parents when the parents want to continue their home life after developing a severe health condition. The medical system for end-of-life care differs by country, but many people want to die in their own home. People with ASD could be caregivers for their family members with appropriate support from medical staff members. Therefore, multiple types of appropriate medical and home care support are necessary.

## Abbreviation Used

ASD: autism spectrum disorder
